# Software's legal future

**DOI:** 10.3389/frma.2022.980744

**Published:** 2022-08-19

**Authors:** Clark D. Asay

**Affiliations:** BYU Law School, Provo, UT, United States

**Keywords:** intellectual property, software, innovation, trade secrecy, patents and copyright studies

## Abstract

The software industry's history is also its future. Its history has been defined by both abundance and scarcity, and its future will be, too. In the 1970s and 80s, perceived software scarcity led U.S. legislators to formally grant intellectual property protections to software creators. Later, a different kind of scarcity—a lack of access to source code—led the founders of the free and open source software movement to flip intellectual property protections on their head in an effort to better promote abundance. That movement proved wildly successful, with today's software industry based on vast amounts of freely available open source software resources that both organizations and individuals collaboratively build. Abundance and scarcity will also define software's future, but in different ways. The abundance that the open source software movement spawned is in the midst of a significant commercial phase. That sometimes means that commercial competitors bring to the table a scarcity mindset that conflicts with the norms that made that movement so successful. Intellectual property concerns at times derail what may otherwise be even greater software abundance. And because so much software is moving into the Cloud, trade secrecy may become the software industry's most important form of intellectual property to the extent the industry abandons open models of innovation. The software industry's growing dependence on artificial intelligence (AI) is likely to contribute to these trends. The software industry is increasingly becoming synonymous with the AI industry, as more and more software companies either rely on AI in running their services or provide AI products to the public. As with all software, these AI technologies are increasingly provided from the Cloud, where trade secrecy is not only possible, but often preferable. But trade secrecy may be even more likely in the AI context because much of the magic in implementing AI systems lies in the know-how to piece them together from available open source software resources, decades-old AI techniques, and data. Hence, to the extent that software and AI technologists spurn open innovation in favor of a scarcity mindset, trade secrecy is likely to become its dominant form of legal protection. The advent of web3 technologies may eventually change some of these trends. But for now, increasing secrecy seems the most likely outcome. I conclude by arguing that this shift to secrecy is likely preferable to other forms of intellectual property.

## Introduction

In 2011, venture capitalist Marc Andreessen opined in a now well-known *Wall Street Journal* editorial that “software is eating the world” (Andreessen, 2011). His point: the modern economy is all about software. Traditional brick-and-mortar companies have either transformed into software companies or fallen to upstarts that successfully made the move. Netflix displaced Blockbuster; Amazon eliminated Borders; Kodak succumbed to the likes of Flickr; and the list goes on. Since his article and true to its prediction, software has only continued its gluttony: our cars, our homes, even our wallets, are all running on or have migrated to software. In fact, for better or worse, it is difficult to find things in the modern world that don't involve code (Somers, 2017).

Such heady times for software were not always certain. When an independent software industry first began to emerge in the 1960s, policy makers worried that developers may be loath to create it without additional legal protections in place (Menell, 1986). In short, software would remain scarce unless developers could effectively monetize it, and policy makers viewed intellectual property protections as crucial to such monetization. Yet others in the free and open source software (FOSS) movement worried that those very protections were inhibiting the industry in its innovative potential, and they took steps to help the software world unshackle itself. Hence, from the software industry's very inception, perceptions of scarcity and abundance have guided the industry in crafting legal rules meant to address scarcity in the pursuit of abundance^1^.

These same concepts are shaping the software industry's future. The FOSS movement has helped create an abundance of readily available software resources (Yeaton, 2011). But that abundance comes with caveats. The movement owes a significant amount of its plenty to commercial contributors—in fact, the FOSS movement is in the midst of a significant commercial phase (Mann, 2006). And while commercial actors have accelerated the FOSS movement in many ways, they also bring to the table a scarcity mindset that sometimes slows, and may ultimately upend, the FOSS movement's open innovation model (Bridgwater, 2019). These undesirable effects may be even more likely as more and more commercial software services move behind the Cloud, where secrecy, not openness, is the norm. Indeed, to the extent that the industry turns its back on open innovation, intellectual property protections—particularly trade secrecy—may regain prominence as tools for addressing a self-imposed scarcity.

The software industry's growing dependence on artificial intelligence (AI) reinforces some of these points. Today, increasingly more software services either rely on AI or provide AI-based products (van Attekum et al., 2019). Yet much of the magic behind these modern-day AI systems lies in the know-how to implement them, rather than the individual components thereof. Indeed, these systems are largely built on well-known AI techniques, FOSS resources, AI-created technologies, and access to increased processing power and data (Asay, 2021). They also largely function from the Cloud, behind closed doors. Hence, to the extent that commercial software and AI providers cling to intellectual property protections, trade secrecy is likely to become the most relevant form for protecting this know-how as well as at least some of the data upon which the systems rely. While other forms of intellectual property protections will certainly continue to play a role, the software industry's AI dependence suggests a future of secrecy.

Of course, that future may not hold for long. So-called Web3 technologies—including blockchain, decentralized, autonomous organizations (DAOs), cryptocurrencies, and non-fungible tokens (NFTs)—promise a future of decentralization, where the masses, rather than governments or a small group of powerful companies, control society's technological landscape. In that future, transparency, not secrecy, is key, and the technology itself, rather than formal intellectual property rights in the technology, may play the most important role in its ongoing development. Even so, that future is not yet here and may never be, even if it is looming on the horizon.

Below, I first trace how perceptions of scarcity and abundance shaped the early software industry and its legal rules and norms. I then look to the software industry's future. I argue that to the extent that the software industry turns its back on open innovation and its spoils in favor of a scarcity mindset, its future is likely to be one of secrecy. I then briefly consider how, normatively, that future of secrecy may be preferable to one dominated by other forms of intellectual property rights.

### The early years of scarcity

At least initially, software's legal status was ambiguous. In the 1960s and 70s, the Copyright Office registered some copyrights in software products even before the copyrightability of software was either judicially or legislatively certain (Samuelson, 2007). Congress dispelled that uncertainty with passage of the Computer Software Copyright Act of 1980, in accordance with the recommendations of the National Commission on New Technological Uses of Copyrighted Works (“CONTU”) (Asay, 2017). By defining “computer programs” and including specific limitations on the rights of copyright owners in computer programs, the Act clearly subjected software to copyright, though it largely left it up to courts to determine the scope of copyright in software (Asay, 2017).

In recommending that software be subject to copyright, CONTU suggested that, if it weren't, copycats could duplicate original software products without incurring the same costs of development, thereby undercutting the ability of software developers to recoup their investments. The result, according to CONTU, would be that few if any parties would be willing to pursue robust software innovation (Asay, 2017).

This might have been particularly true in light of another development in the software industry that CONTU highlighted: software developers were increasingly not able to recoup their costs of development from hardware sales because software had become its own market. Previously, hardware and software developers were often the same party, with software being developed and customized for a particular hardware product. But that had changed. Software and hardware developers were now often different parties. Consequently, many software developers could no longer recoup their costs of development through the sale of hardware products. Instead, they needed to sell their software, and CONTU saw copyright as an important part of them being able to do so (Asay, 2017).

By some accounts, copyright played exactly that role in subsequent decades. The software industry began to boom, and commentators pointed to copyright protection as playing at least a “nontrivial role” in spurring that boom (Samuelson, 2011). While other factors certainly influenced this growth, copyright appears to have motivated at least some, and perhaps many, developers to create socially useful software products.

Patent protection for software followed a similar timeline as that of copyright. And according to some accounts, it played a similar role in encouraging software innovation in these early years (Con Diaz, 2019). In the 1960s and 70s, patenting software, on its own, was an uphill battle. The United States Patent Office appears to have rarely granted patents on software alone, even issuing formal guidelines prohibiting such patenting. Despite this, some point to instances of the Patent Office issuing software patents during these early days (Quinn, 2014). Nonetheless, during this time the Supreme Court ruled against at least some patents on software, finding certain software products to be outside the scope of patentable subject matter because, on their own, those products were simply mental abstractions aimed at performing unpatentable ideas and mathematical equations (Quinn, 2014).

That attitude began to change in the 1980s, with the Supreme Court deciding that at least some software innovations could be patentable subject matter (Campbell-Kelly, 2005). And by the 1990s, several additional judicial decisions further established the patentability of software. The numbers of software patents, unsurprisingly, grew significantly during these decades (Bessen and Hunt, 2007).

According to some, software patenting was a key driver in pushing the software industry forward during this time (Campbell-Kelly, 2005; Quinn, 2014; Con Diaz, 2019). Similar to copyright, patents provided software developers with a means of recouping their costs of software development. In fact, according to some commentators, patents were an even better mechanism for doing so for several reasons. First, patents are not subject to an independent creation defense as with copyright (Campbell-Kelly, 2005; Mossoff, 2013). With copyright, competitors could study the copyrighted software program, figure out its functions, and then feed those parameters to their developers with instructions to create a similar program Mossoff, 2013). The newly created program would not violate the copyright protections in the original program. A patent on the same program, on the other hand, could be used to prevent such duplication, so long as the patent claims covered what the competitor had copied into its own program. Second and related, patents can protect inventive ideas, whereas copyright protection only covers the expression of ideas (Quinn, 2018).

Trade secrecy was also an available legal protection for software during these early years. But it came with significant drawbacks. Trade secrecy provided developers with protection against others obtaining access to their software through improper means or a breach of confidence (Fromer, 2019). In order to qualify as a trade secret, the software must not be generally known or readily ascertainable, possess independent economic value, and be subject to reasonable precautions under the circumstances to protect its secrecy (Fromer, 2019). Early on, particularly in the face of doubt as to whether copyright or patents applied to software, some software innovators relied on trade secrecy as their primary form of legal protection (Campbell-Kelly, 2005). They subjected their customers to non-disclosure agreements and other restrictions that were meant to prevent their software secrets from becoming known to others and thereby losing their trade secret status (Id.).

But such protection was always tenuous. If the software developer wished to sell their product on the open market, their trade secrets may be obvious once distributed or readily ascertainable through reverse engineering, thereby extinguishing trade secret protection (Hrdy and Sandeen, 2021). Consequently, for many products, maintaining trade secrecy was incompatible with selling them on the market.

Furthermore, trade secrecy's available remedies are in some ways inferior to those that copyright and patents provide. For example, even in cases where trade secrecy could be maintained while selling the product on the market—through non-disclosure agreements, releasing the product in object code only, or otherwise—a savvy hacker may still discover the secrets and share them with the rest of the world. The trade secret owner in such a case would have a cause of action against that particular hacker, but typically would be out of luck vis-à-vis the rest of the world (Scherf and Gering, 2021).

Copyright and patents, in contrast, would still protect the author against anyone making use of the software, subject to certain exceptions. Furthermore, trade secret injunctions often only last as long as it would have taken the appropriator to develop the information themselves, whereas copyright and patent injunctions, at least at the time, were much more robust (Dole, 2011). In short, while trade secrecy was certainly an option for software developers, copyright and patents had several relative advantages early on (Samuelson et al., 1994).

Hence, early in the software industry's history, a scarcity mindset predominated. By the 1980s, courts, Congress, and innovators had recognized patents and copyrights as important tools for spurring software innovation. Without copyright and patent protection, would-be software developers may never pursue socially optimal amounts of software innovation and thereby “promote the progress of Science and the useful Arts,” the constitutional basis for granting such protections. This may have been particularly so in light of trade secrecy's significant limitations in terms of both the scope of protection and the available remedies. Copyright and patents, by providing developers with a more robust means by which to recoup their development costs, arguably motivated at least some, and perhaps many, to pursue software development. But whatever their role, the software industry experienced significant growth during this time.

### The free and open source software movement's abundance

Yet even as courts and Congress recognized copyright and patent protections for software, another movement was afoot. Starting in the 1980s, some software developers began to voice frustrations about their inability to improve software products licensed from third parties (Neary, 2018). Their inability to do so was because of intellectual property protections. For instance, if a software developer ran into a malfunctioning printer, the terms of the intellectual property license agreement applicable to the malfunctioning software often prohibited them from fixing the machine by tinkering with its software. Furthermore, the users typically had neither actual nor legal access to the source code necessary to perform the fix. Legally, they were stuck, and the only way forward was to seek permission from the rights holder, a cumbersome process that typically resulted in denial.

As a result, some of these early software developers took matters into their own hands. They started what came to be known as the “free and open source software” movement (FOSS) (Neary, 2018). This movement has a complicated history, and it is not the purpose of this article to review that history in full. For our purposes, the movement did several important things. First, it developed a suite of intellectual property licenses that enabled, rather than prohibited, the types of uses (and others) that typical intellectual property licenses prohibited (Tozzi, 2016). These licenses generally allowed others to modify and use the software subject to them in whatever way users saw fit, so long as certain conditions were satisfied. *One* of the most important ones, at least early on, were so-called “copyleft” provisions, which required users of the licensed software to grant the same rights to any others to whom they distributed the software (Free Software Foundation, 2018). The idea was to spread norms of openness and freedom by conditioning use and further distribution of the software on granting others the same rights. These legal innovations proved successful by any definition of the word. Parties began adopting these licenses for many of their software projects (Neary, 2018). And while some parties, particularly commercial actors, showed reluctance to use software subject to such terms, the enticement of otherwise freely available software was often enough to get many parties over the hump—at least eventually (Neary, 2018).

Second and related, parties within this movement began to collaboratively develop software resources subject to these licenses as alternatives to dominant proprietary solutions. Perhaps the best-known example is the Linux kernel, released under the General Public License. Linux was meant to provide a FOSS option for operating system software that parties could use instead of dominant proprietary options from the likes of Microsoft and IBM (Neary, 2018). Today, Linux is the backbone of much of the computing world (Finley, 2016). But even beyond Linux, FOSS developers began to provide FOSS solutions that steadily displaced proprietary solutions along the entire software stack because of their source code availability, reduced costs, and, in many cases, technical superiority (Ahlawat et al., 2021).

In fact, a major premise of the FOSS movement is that open, collaborative innovation is far superior to a siloed, closed approach (Raymond, 2000). As one of the early FOSS leaders once articulated, “given enough eyeballs, all [software] bugs are shallow.” (Id.) And as the FOSS movement began to take off, it became increasingly clear that the road to greater software abundance, both in terms of quality and quantity, was through open innovation.

Today, the FOSS movement's successes speak for themselves. Every major technology provider both uses and contributes to FOSS projects. Early hesitancy to using FOSS because of licensing terms has been replaced with near dogma that every software solution should start, and often end, with FOSS (Szulik, 2018). FOSS is in every computing device, and nearly every type of software problem has at least one, and often many, FOSS solutions (Id.). While proprietary software development still occurs, it typically does so in the shadow of FOSS.

The software industry's embrace of FOSS has accelerated its pace and scope of innovation. The FOSS movement's abundance mindset has led to a significant surfeit in both the quality and quantity of software resources that parties ranging from individual developers, to start-up companies, to large, multinational companies regularly use and to which they contribute. And while a FOSS approach may not always make sense for a particular scenario, it has largely become the software industry's go-to approach.

Furthermore, studies have shown that many parties that participate in the FOSS movement are motivated by things other than intellectual property rights (Schlaefli, 2014). This might be obvious, but remember that the primary reason for granting intellectual property rights in software was that without them, parties may not pursue software innovation. In many circumstances, at least, that theory simply isn't true. Parties pursue FOSS development for all kinds of reasons, including intrinsic motivations such as desires to be creative and to contribute that creativity for the greater good (Id.). Of course, much software development occurs as part of peoples' employment, and the availability of intellectual property rights may certainly motivate many of these employers in funding their employees' software development activities (Asay M., 2018). Yet many such employers are willing to give up those rights in exchange for being able to use and contribute to FOSS projects. Indeed, some companies have even publicly pledged their intellectual property rights to help further the FOSS movement's ascension (Contreras, 2015).

In sum, early on a scarcity mindset motivated courts and Congress to provide for intellectual property rights in software. And at least some, and perhaps many, software developers may have been loath to pursue software innovation without those rights in place. Yet with the FOSS movement, an abundance mindset came to triumph in the software industry's evolution. Of course, parties still continued to register copyrights and obtain patents in software products during that evolution, particularly large technology companies that viewed these protections as key assets even as they continued to further adopt and contribute to FOSS projects. Yet other parties used those same assets to promote the FOSS movement, turning a scarcity mindset on its head to promote a vision and realization of software abundance.

### The software industry's future of abundance and scarcity

The FOSS movement's abundance has not eliminated scarcity in the software industry. Instead, in the modern age, it is intersecting with new forms of it. First, the FOSS movement going mainstream means that a scarcity mindset is increasingly in play as commercial actors compete with one another. That mindset often conflicts with and complicates the otherwise rosy story we might tell ourselves about the FOSS movement's triumphs, particularly as more software moves into the Cloud. Second, AI has changed the game. The software industry today is in many ways coterminous with the AI industry, because nearly all software developers use forms of AI in their software solutions. But there is a scarcity of human know-how capable of using and deploying today's AI technologies, which typically operate from the Cloud and are largely based on FOSS resources, well-known AI techniques, and greater access to data and processing power. This all portends a future in the software and AI industries where trade secrecy reigns supreme.

## The FOSS movement's commercialization

The FOSS movement has always included commercial players. In fact, one of the early debates within the FOSS community was how the movement and commercial entities should coexist (Stallman, 2021). Those debates led to divisions among many early FOSS leaders about which FOSS licenses should predominate. Some believed that commercial support was key to spurring the FOSS movement forward. These leaders often favored more permissive licenses that would reduce the concerns of commercial parties and thereby encourage their participation in using and contributing to FOSS projects. Others believed that the movement should not cater to commercial interests. Instead, the FOSS movement should stick to first principles and require anyone that uses FOSS to also adhere to those principles. These parties thus favored licenses that required users of the software to contribute back any modifications they made to the software under the same license terms, regardless of whether such provisions scared away potential commercial contributors (Id.)

Whatever might be said of those early licensing arguments, those in favor of significant commercial involvement ultimately won the day (Robles et al., 2019). That may or may not be because of the triumph of more permissive FOSS licenses, though a good amount of evidence suggests that more permissive licensing has coincided with growing commercial adoption of FOSS (Johnson, 2021). What is clear is that commercial adoption of and contributions to FOSS projects have grown astronomically over time (Robles et al., 2019). And companies that develop and distribute FOSS as their primary commercial activity have surged, too, even as difficult questions persist about the best ways to make such commercial endeavors successful (Solomon, 2020). Be that as it may, the FOSS movement is now in significant part a commercial movement. Most parties that contribute to FOSS projects are paid to do so, and most code contributed to FOSS projects comes from commercial actors (Volpi, 2019). Of course, non-commercial parties still found, contribute to, and participate in FOSS projects, though their interest in participating still often has a commercial dimension (Wachal, 2019). But the commercial world, with all its vast resources, has gone all in on FOSS.

That has created some tensions. While commercial players have recognized the value of both using and contributing to FOSS projects, they still exist within a competitive environment. For many technology companies, part of responding to that competition centers on maintaining robust intellectual property portfolios. For instance, for many technology companies, a key strategy in responding to commercial competition has been to obtain ever-more numbers of patents (Eveleth, 2019). Technology companies often use these large portfolios primarily as a defensive mechanism—they build large patent portfolios to help ward off threats from their competitors. Some have likened this patent strategy to the Cold War, where superpowers built up nuclear arsenals, not necessarily to use the weapons, but instead to rely on the threat of using them to keep their competitors at bay (Harrington et al., 2017).

But large technology companies also at times use their portfolios offensively—to thwart a competitive product, to extract rents from a competitor, or to play bully ball with industry upstarts (Duhigg and Lohr, 2012). These types of offensive uses reinforce the defensive purposes in that both motives contribute to a drive to obtain patents. The result has been large accumulations of software patents, primarily by the biggest players (Roberts, 2021). But startups and the like also frequently acquire patents, both to protect themselves against competitors and to signal to funders that they are innovative (Lee, 2017). Even some FOSS companies have begun to acquire patents for defensive purposes, believing that doing so is necessary given the high rates of patenting in the technology sector (Broersma, 2002).

Trade secrets are another asset type that companies in a competitive environment often seek to protect. In fact, trade secrets can be some of the most important assets a company possesses (Linton, 2016). This may be so for several reasons. First, trade secrecy can protect information beyond what other forms of intellectual property cover. Patent law includes specific exceptions to patentable subject matter, and these have expanded over time (Lemley and Zyontz, 2021). Trade secrecy can provide protection to things that these exceptions leave outside the scope of patentable subject matter (Simon and Sichelman, 2017). Second, trade secrecy can also last forever, so long as the conditions of trade secrecy are met. Conversely, other forms of intellectual property protection typically expire after a set period of time. Finally, trade secrecy is often cheaper, though taking reasonable precautions to protect one's secrets can entail significant costs in the cumulative (Khoury, 2014). But compared to patents in particular, which entail a costly prosecution process, trade secrecy's costs largely boil down to simply maintaining information as a secret (Schechter and Thomas, 2004).

Many software companies are in an excellent position to reap trade secrecy's advantages because, in the modern age, increasingly more of their services are Cloud-based. In fact, in the modern age, the “as a Service” revolution includes nearly every type of software resource—today, very few software products are *not* provided from the Cloud (Ramachandran and Linthicum, 2020). This means that companies' software never need be distributed outside of the company. As a result, companies are in a better position to keep information relating to their software, including the source code, secret. Of course, the public-facing aspects of their services are not protectable as trade secrets because they are observable upon the public's use of the service. But companies can maintain other important aspects of their services as trade secrets precisely because the services are Cloud-based.

Finally, many technology companies may wish to protect their copyright interests in their software technologies. Of course, in many cases software companies find it palatable to grant other parties access to their copyrighted materials—the history of FOSS is replete with examples thereof. But in other cases, companies may wish to withhold their copyrighted materials from their competitors because of the perceived advantages that those copyrighted materials provide those companies (Westgarth, 2019).

The conditions of using and contributing to many FOSS projects often conflict with companies keeping their intellectual property rights unsullied. Many FOSS licenses include either implied or express patent licenses (Gatto and Koo, 2018). This means that using and modifying FOSS materials so licensed may impact a company's patent portfolio. FOSS licenses all include licenses to copyright, resulting in similar concerns with respect to copyright. And trade secrecy is out of the question when companies are required to make their source code available to the public in accordance with certain copyleft licenses. Furthermore, FOSS projects often include contribution agreements. These are the terms that apply to a party's contributions to a particular FOSS project. While they often mirror the applicable FOSS license, they also frequently include additional terms, including requirements that contributors license (or in some cases assign) their relevant intellectual property rights to the FOSS project and its users.

Because of these possible effects, savvy companies are careful in their uses of and contributions to FOSS. They often implement processes for reviewing all uses of and participation in FOSS projects (Asay, 2013). Most sophisticated companies require multiple levels of approval before an employee can participate in a FOSS project or use FOSS materials in the company's products or services (Id.). And many conduct regular audits to determine what FOSS is in use within the company. Based on these reviews and audits, companies often deny participation by their employees in FOSS projects and use of FOSS materials that may negatively impact their intellectual property portfolios (Id.).

The result is that intellectual property concerns—or a scarcity mindset—often prevent even greater abundance in the form of increased commercial use of and contributions to FOSS projects. Of course, this is all a matter of degree rather than kind. For now, commercial software entities still seem largely committed to using and contributing to FOSS projects, both as a competitive advantage and necessity. There probably is no going back from FOSS, at least all the way to a pre-FOSS world. But intellectual property concerns do get in the way, at times, of even greater collaboration and growth in the FOSS movement and the software industry more generally.

This seems like a nearly intractable problem. Commercial competitors, perhaps ineluctably, view the world with a scarcity mindset: another party's gain is the primary party's loss, and it's the goal of perhaps all companies to always be on the road to more gain. Public companies, with their shareholders, are in many ways bound to pursue that path. And while growing the pie for everyone might be a nice (and sometimes true) soundbite, the reality is that much of the time, commercial competitors simply don't abide by it (Tian and Smith, 2014).

In fact, the FOSS movement's commercialization has reinvigorated early debates about the role of commercial entities in the FOSS movement. As commercial entities have become more involved with many FOSS projects, that involvement has at times led to those entities taking on formal leadership roles within those projects (Traverso, 2021). Furthermore, their significant contributions to many FOSS projects are often the primary drivers of innovation within those projects, and that reality provides them with de facto control of the projects even outside of their formal positions in the projects' governance regimes (Lifshitz-Assaf and Nagle, 2021). This outsized influence has in some cases led to friction with the non-commercial leadership of various FOSS projects, with calls to jettison the outsized influence of some of those commercial actors (Mih, 2021). In short, while many FOSS projects have benefited greatly from commercial involvement, that involvement has also resulted in formal and informal constraints on those projects' leadership and future directions.

Commercial entities' involvement has also reinvigorated debates about preferred licensing terms, particularly as more and more companies provide their services from the Cloud (Mih, 2021). For instance, many FOSS licenses do not require parties to contribute back their improvements to the community unless those parties distribute the software (Tozzi, 2020). Or in other cases, permissive licenses simply fail to require users to share their changes to the software with the community at all (Id.). Because so many commercial entities provide their services from the Cloud, they are able to avoid distributions that would trigger sharing obligations. And in the case of licenses that don't require sharing their improvements, the Cloud provides a perfect cloak for many of their innovations. As a result, some within the FOSS community have called for more FOSS to be licensed under terms that would require commercial entities to share their changes to FOSS even when it is provided as a service (Id.). But so far, the industry, by and large, has not moved in that direction.

Hence, while the FOSS movement has created a significant amount of software abundance, the commercial world's involvement with creating that abundance has become a double-edged sword. The FOSS movement's successes owe significantly to commercial participation. But that participation also means a scarcity mindset is ever present, and perhaps growing, as part of that movement. It can also mean significant constraints on the project's future direction in light of the commercial actors' influence within the projects. In all likelihood, the benefits of commercial involvement, in the form of abundant software resources, outweigh the costs, which primarily come in the form of a pumping of the breaks on the FOSS movement's acceleration. For now, at least, that tradeoff seems to be worth it.

But one can imagine a world where that is not the case. As more and more software moves into the Cloud, commercial competitors seem ever more likely to protect many of their innovations as trade secrets. In fact, as mentioned above, there is already growing concern that many commercial entities are not contributing back nearly enough to the FOSS projects from which they profit. Instead, they often take what they need while keeping many of their improvements secret, behind the Cloud. Such maneuverings may not kill FOSS development off completely—commercial entities still significantly contribute to FOSS projects and are likely to continue to do so, particularly in areas that are less commercially strategic than others. But these shifts do point to an end of a golden age of open innovation in the software industry. The rise of AI, discussed next, may reinforce such trends.

## Artificial intelligence's ascent

AI is affecting the software industry in at least four important ways. First, software companies are increasingly AI companies, and vice-versa. While not all software providers are AI providers, increasingly more of them use AI in some form in providing their products. This means that, today, more and more software companies provide AI services, or services that rely on AI. Second, as with all software, AI services are largely provided from the Cloud (Uslu, 2021). Third, AI technologies are largely built from FOSS resources, well-known AI techniques, and improved access to data and processing power (Cronin, 2016). In short, much of the magic of modern-day AI implementations lies in the know-how necessary to stitch them together from these resources, rather than any individual components thereof. Finally, AI tools have sped up software development in a number of ways, even in some cases rendering software obsolete. These realities reinforce the likelihood that trade secrecy, more than other types of intellectual property, will reign supreme in the software industry going forward. As I discuss later, this development may slow software abundance some, though it is likely preferable to other forms of artificial scarcity that patents and copyrights provide.

### The software industry's AI and cloud transformations

Today, nearly every software company is in some ways an AI company because software services depend on AI, and vice-versa. This doesn't mean that all software companies provide AI services, though there are many that do (Ohnsman and Kai, 2021). Instead, many software companies use AI tools to build software or provide their services or both, even when they don't provide AI products directly to the market (Rangaiah, 2020).

For instance, AI tools exist to automatically produce different software resources, including software code and interfaces. These tools can speed up the coding process in many cases. Furthermore, many software services incorporate AI as part of the service, even when the service itself is not strictly the provision of AI. Netflix, for instance, uses AI to recommend new content, while Facebook uses AI to optimize news feeds. And while there are some corners of the software industry where AI may not be as relevant as in others, ultimately that is unlikely to remain so. AI will eventually touch every nook of the software industry, even if it hasn't already.

Furthermore, as with software more generally, AI-based services largely operate from the Cloud. This includes both the provision of AI to customers and software services that are built using AI tools or that incorporate AI in providing the service. This merger between AI and Cloud computing is only likely to grow, as both technologies can enhance the effectiveness of the other.

This all means that many of the innovations that are happening or will happen in the software industry going forward will relate to AI. It also means that they will primarily be provided from the Cloud. Both of these realities reinforce the argument made above that trade secrecy will be the most critical form of legal protection in the industry going forward.

Too see why, consider the following: the FOSS movement has created vast amounts of software resources, freely available and collaboratively maintained by a worldwide force of developers and companies, as discussed above. This has pushed competitive innovation up the software stack. That is, much of the common infrastructure upon which everyone relies consists of various FOSS projects (Lifshitz-Assaf and Nagle, 2021). Companies make their mark, so to speak, by building goods and services on top of that infrastructure (Wessell and Ng, 2015). And in the modern age, those goods and services are often either AI products or services that rely on AI.

But there is a world-wide shortage of people who can successfully implement AI systems (Gehlhaus, 2021). Successful AI deployment often mostly has to do with knowing how to piece together an AI system from available resources (Marr, 2018). As mentioned above, many of the software pieces necessary to run the AI system are freely available FOSS, including much of the relevant infrastructure. Similarly, the deep learning AI techniques that many companies wish to implement as part of their services are in the public domain—and have been for decades (Anyoha, 2017). Access to data, another key component of successful AI systems, is rising, though barriers in many cases remain. In fact, many, perhaps most, attribute the rise of AI to increased access to data and processing power, rather than any revolutionary change in the underlying AI techniques (Asay, 2020). Yet despite the general availability of these different components of AI systems, the know-how to piece them together is significantly lacking (Metz, 2017). Companies are in fierce competition to secure this limited resource, with salaries for AI specialists skyrocketing as a result (Id.).

Hence, this know-how, rather than software resources or even the relevant AI techniques, is a new form of scarcity in the modern software industry. And that means that trade secrecy, more than other forms of intellectual property, may be the most important form of intellectual property in the software industry going forward. This is so for at least several reasons.

First, companies' competitive edge in such an environment will center on things that trade secrecy is best suited to protect. As mentioned above, it is not the public domain AI techniques that provide the competitive edge, but rather the ability to successfully implement them. Companies can't patent those techniques, even if they can and do obtain narrower patents on particular implementations thereof. But the tacit knowledge surrounding how to implement and carry out a successful AI implementation is likely even more valuable and something that trade secrecy is best suited to protect.

Indeed, the reality that these AI implementations frequently occur behind closed doors as part of a Cloud-based software offering means that companies can often shield their AI trade secrets from the public's view. In fact, this ability may be a significant reason why parties forego seeking at least some patents on their narrow AI implementations, because withholding that information from the public, possibly in perpetuity, is often more valuable than a limited-term patent on a piece of that know-how (Gibson and Buchman, 2021).

Furthermore, much of the competitive advantage in AI implementations centers on data: AI systems are only as good as the data fueling them. But such data is neither patentable nor copyrightable, at least in a way that provides much protection. Trade secrecy, however, can protect such data, providing yet another reason why trade secrecy may be the software industry's most important form of intellectual property going forward.

Finally, copyright may become increasingly irrelevant in the software industry, at least in its traditional utilitarian role. Aside from its futility in protecting the data that fuels modern AI systems, copyright also seems feeble with respect to software. Copyright certainly applies to software. But as discussed, FOSS resources make up much of the infrastructure fueling modern AI systems, and copyright plays quite a different role with respect to FOSS.

Trade secrecy is also likely to prove important because it will be the center of many disputes as AI specialists move from one company to the next. As companies fight over available AI talent, that competition is likely to result in significant employee migrations between companies. As they do so, trade secret fights are likely to result because the know-how that an employee takes from one company to the next is more likely to be trade secret information than other types of intellectual property assets, for the reasons discussed above.

In fact, we've already started to see high-profile cases along these lines. In 2020, Anthony Levandowski, a former Google executive, was sentenced to 18 months in federal prison for stealing trade secrets from Google's Waymo and selling them to Uber Technologies (Statt, 2020). These trade secrets concerned AI relating to self-driving cars. Levandowski is considered one of the world's foremost experts and pioneers in this field, and parties such as Uber were willing to pay top dollar for his expertise. Unfortunately for Levandowski and Uber, that expertise crossed the line into trade secrecy.

### AI's transformation of software development

Another reason trade secrecy is fast becoming the software industry's most important form of protection is AI's role in helping create software. Today, freely available AI tools can help software developers obtain helpful software code with little input from those developers (Loukides, 2020). AI tools exist for creating interfaces and source code, which help speed up software development (Choudhury, 2019). Low coding, another form of minimalistic software development, also enables even non-programmers to develop software applications with little technical know-how, though those with technical acumen may be needed to bring those applications up to snuff (Sacolick, 2020). Overall, these types of tools facilitate more rapid development of software, thereby speeding the rate of software innovation. And it's a trend that is likely to grow.

Yet it is unclear to what extent copyright and patents apply to AI-created software inventions and works of authorship. Both copyright and patent law appear to require human authors for rights to issue (Richey and Mammen, 2019; Krumplitsch et al., 2021). But with AI, it is increasingly unclear whether the AI agent or the human authors are primarily responsible for whatever the AI tool produces. Of course, in most cases some human involvement is still necessary, and that involvement will likely be enough to count as human authorship for purposes of both copyright and patent law (Fjelland, 2020). But the human's involvement may still influence the scope of the author's rights in the resulting work. For instance, if a human's contribution is a minimal amount of creativity, that creative expression may be what the human has a copyright interest in, rather than AI-provided creativity in response to the human input. Similarly, if a human feeds an AI system a patentable idea that the AI system expands upon, arguably the human is only the author of whatever patentable idea they supplied, not the entirety of what the AI system ultimately produced.

Furthermore, in some cases one can imagine a human author having no rights in the work product of AI systems, even when their input guided that work product. For instance, if a human author's sole contribution is to provide a general idea as to what the software should do, and the AI creates software that implements that idea, arguably the human should not have any copyright interest in the result because all they contributed was an idea, which copyright does not protect. As for patents, the idea may be so abstract as to fall outside the scope of patentable subject matter (Morris, 2018). Furthermore, the AI tool may be responsible for whatever novelty or non-obviousness inheres in the AI-created solution. In other words, the supplied idea may lack novelty and non-obviousness, even if the AI-created solution satisfies both requirements.

In all of these cases, trade secrecy may be the best intellectual property solution. While copyright and patents may still apply to some extent, the complexities discussed above will often make trade secrecy a better route. Furthermore, that many of these solutions operate from behind closed doors as part of Cloud-based solutions makes trade secrecy even more appealing. Finally, the software solutions that these AIs create may often lack a competitive edge on their own; instead, their value inheres as a small piece of a larger AI implementation. As discussed above, often it is the know-how to stitch all the individual pieces together, rather than the individual pieces in isolation, that are valuable. And trade secrecy will often be the most relevant form of protection for such know-how.

At least in some circumstances, AI also renders software development obsolete. In the deep learning context, for instance, some have argued that because AI directly creates and implements the relevant algorithms, AI increasingly displaces the need for additional software development (Morris, 2018). The algorithms and data are what is key in these systems, things that trade secrecy, more so than any other form of intellectual property, is best equipped to protect.

## The ascent(?) of Web3 technologies

Of course, the future is always uncertain, and trade secrecy's reign may be short-lived to the extent that the promises of Web3 come to fruition. It isn't simple to pinpoint what those promises are—indeed, one of the main gripes with Web3 is difficulty in defining it or identifying concrete use cases (Nield, 2021). But at an abstract level, the promise of Web3 is decentralization—or, in the parlance of this Article, greater abundance for everyday people. In such a world, no longer are governments or large, multinational companies the gatekeepers to currencies or the technologies that we use. Instead, Web3 may mean that everyday people have greater say in and control of the technologies that they use (including currencies). And those everyday people get to reap more of the monetary benefits of those technologies as well. They do so by owning tokens, both fungible (think cyptocurrencies) and non-fungible (think NFTs), in a particular piece of technology or system. Those tokens allow them to do any number of things depending on the underlying system. But the key in all cases is greater participation, influence and, potentially, financial upside. Importantly, blockchain technology is the backbone of Web3. In its simplest sense, a blockchain is a distributed database whose distributed nature helps ensure that the database accurately reflects whatever transactions have been recorded on the database. It is “trustless” technology that helps disparate parties transact with one another by ensuring that all involved meet whatever obligations they enter.

We need not get further into Web3's technical weeds. Indeed, they are still being determined as Web3's advocates seek to chart the future. Important for our purposes is that Web3's decentralization thesis may portend a very different legal future for software than the one previously discussed. After all, to the extent that the current set of tech monoliths are displaced with decentralized power structures, commercial actors' preferences for intellectual property protections, particularly secrecy, may succumb to a collective desire for transparency. Indeed, in important respects blockchain technology depends on transparency to accurately reflect the state of a particular distributed database. Furthermore, it may be the case that decentralized systems are simply less concerned with intellectual property protections than they are with the state of the underlying technology itself.

For instance, the key to cryptocurrencies such as Bitcoin is that the distributed blockchain agrees on who owns what, not the intellectual property rights in the technology *per se*. NFTs, another important piece of Web3, similarly derive their value through technological agreement rather than intellectual property rights. Of course, intellectual property rights may still play a role in incentivizing parties to develop the software and associated technologies underlying Web3. But much of that development is based on FOSS, where intellectual property rights play quite a different role, as discussed above. Furthermore, decentralized decision-making may place less emphasis on the accumulation of intellectual property rights, the typical approach of large, centralized corporations, and more on funneling resources into technological development that expands a particular product's network and thus it's value. In a sense, Web3 may free FOSS development from its current commercial masters and get it back to its roots—software abundance for the benefit of all.

Yet as eager as Web3's advocates are for the present to be the future, Web3's future remains murky. Hence, while this decentralized pipedream may eventually become reality, we simply aren't there yet. Time will tell to what extent Web3's vision comes to fruition. For now, trade secrecy is likely to remain software's legal future.

### The advantages of a “Secret” future

On its face, this future of secrecy may portend ill. After all, to the extent that secrecy displaces the highly successful FOSS movement and its open mode of innovation, secrecy's ascent may turn back the clock, so to speak, to an era when software innovation was not nearly as successful as it is today. The FOSS movement has been and continues to be the engine of some of the most significant technological advances in the modern age. It would be a shame if the software industry turned its collective back on the lessons this movement has taught: that open innovation leads to more abundance than imposing artificial scarcity.

Yet secrecy can coexist with openness, and that seems to be the software industry's most likely future. Despite rumblings of discontent, open innovation is almost certainly here to stay, even if it may sometimes stall with certain projects or in certain corners of the software world. As discussed, open innovation seems likely to continue to push innovation higher and higher up the software stack. Higher up on that stack, secrecy may be the prudent option for companies with respect to non-commoditized pieces of their products and services, while FOSS can continue to work its magic in creating widely available infrastructure resources. Competition and secrecy can continue at the top of the stack, while openness and collaboration continue to push that competition upwards.

For a number of reasons, trade secrecy may be preferable to other types of intellectual property rights when zeroing in on the top of that stack. For instance, patents, once granted, often take on lives of their own. Companies often obtain them as a matter of course, with no clear, immediate objective in mind. Yet once those assets are obtained, it becomes somewhat foolhardy for companies to forego trying to monetize them in some form or another. The patent holder may thus pursue activities aimed at realizing some value from its patent investments, either by directly asserting their patents against third parties or outsourcing that work to others (Asay C. D., 2018). This can be particularly wasteful when the patents asserted are ambiguous, a characteristic that many technology patents exhibit (Bessen and Meurer, 2008).

Some might argue that patents are preferable to trade secrecy because patents force disclosure of information relating to the patented invention, whereas the whole point of trade secrecy is to keep that useful information hidden from the rest of society. However, there are longstanding, significant concerns that technology patents in particular don't disclose much useful information (Bessen and Meurer). Furthermore, there are several reasons why other technologists often avoid actually reading relevant technology patents (Roin, 2005). Furthermore, as Mark Lemley has argued, trade secret protection often plays a disclosure role itself—by providing trade secret owners with some protections, trade secret protection encourages them to engage in transactions wherein they disclose their secrets to others (Lemley, 2008).

Furthermore, trade secrecy is a relatively weak form of protection that can be extinguished once the cat's out of the bag. It is also weak in that the line between general skill and knowledge—which can't be protected—and secret information is often difficult to draw. This may make at least some trade secret claims less likely to materialize since the risks of wasting valuable resources on unsuccessful suits is higher. This is not to say that trade secret owners should not pursue valid trade secret claims against misappropriators. But it is to say that the characteristics of trade secret protection may reduce the risk of frivolous, wasteful lawsuits, whereas the characteristics of many patents push in the opposite direction. And fewer frivolous lawsuits are a benefit to society.

Indeed, a final, related comment about other forms of intellectual property when compared to trade secrecy is that those other forms have significant lifespans. Patents last 20 years from the filing date, while copyrights continue the life of the author plus another 70 years, subject to a number of other permutations. In both cases, the lengthy term of protection often increases the likelihood of frivolous, wasteful lawsuits that impede rather than promote innovation (Love, 2013).

Of course, theoretically, at least, trade secrecy can last forever, and in some cases is maintained for long periods of time. Hence, when compared to patents and copyright on this point, trade secrecy may seem inferior. But the reality is that parties in most cases only spend the resources necessary to maintain trade secrecy so long as doing so is required to protect some economic interest. Hence, once secrets are no longer valuable, they are much more likely to lose trade secret protection because the owner will stop spending resources to keep them secret. This means that trade secrecy, more than copyright or patents, naturally aligns itself with the rational term of protection. And that alignment helps mitigate anticommons and holdup concerns that may otherwise arise.

Hence, overall a world of partial secrecy seems preferable to one where other forms of intellectual property, particularly patents, reign supreme. Realistically, a mixed world of some trade secrecy and openness is the most likely future. In that future, trade secrecy can serve its purpose and then be gone. That seems like a desirable outcome compared to the world of copyright and patents, which tend to persist and can continue to introduce hurdles long after their utility has passed.

## Conclusion

The conclusion that trade secrecy is software's foreseeable legal future is not meant to suggest that other forms of intellectual property will be irrelevant in the software industry going forward. This is particularly so given the ongoing commercialization of the FOSS movement, as discussed above. Parties continue to file for and obtain large numbers of software patents (Millien, 2021). And they are likely to continue to do so even if FOSS continues to predominate. The patents they obtain may be narrower—and thus less valuable—than in the past for several reasons, including Supreme Court decisions that have effectively made obtaining broad software patents more difficult (Lee, 2014). Furthermore, while the FOSS movement uses copyright in a different way, copyright protection is still the foundation for spreading FOSS norms. And while FOSS is the backbone of the software industry, proprietary software development still occurs in some areas, meaning that copyright may still play its traditional utilitarian role in such cases. Trade secrecy isn't everything, even if, in the modern software industry, it is fast becoming the most important thing.

It's also possible, even likely, that the software industry will evolve over time in way that trade secrecy's importance ultimately wanes. As discussed above, Web3 may become a widespread reality, meaning that the importance of intellectual property rights, including trade secrecy, will change significantly. Aside from that, the current worldwide talent shortage in AI talent is likely to change as markets adjust to that shortage. An eventual infusion of AI talent may create abundance where scarcity once was. Furthermore, while the AI industry currently relies on well-known public domain techniques, it may eventually find its way into more general forms of AI. If it does, a significant patent race may ensue. In such a scenario, trade secrecy may find itself displaced as the software industry's most important form of protection, at least for a while.

Finally, while the FOSS movement's mode of software production may be firmly entrenched now, there is no guarantee that it will remain so. Particularly as more and more FOSS becomes commercialized, the software industry may revert more forcefully to its scarcity mindset, in contravention to the ideals espoused by Web3 advocates. This reversion would seem like an irrational development in light of the FOSS movement's successes. But markets certainly don't always behave rationally. And if the software industry were to go that route, copyright may regain its role as a predominantly utilitarian incentive, rather than its current one of fostering FOSS norms.

But at least for the foreseeable future, trade secrecy will be the software industry's most important form of protection in light of that industry's current set of realities. Those realities include an abundance of software resources and public domain AI techniques, but a scarcity of the know-how to use them in a data-driven world. For those scarce resources, trade secrecy is the most relevant intellectual property protection, particularly in a Cloud-centered landscape. And relatively speaking, that world of secrecy high atop the modern software stack is almost certainly preferable to other intellectual property forms for the reasons discussed above.

## Data availability statement

The original contributions presented in the study are included in the article/supplementary material, further inquiries can be directed to the corresponding author/s.

## Author contributions

The author confirms being the sole contributor of this work and has approved it for publication.

## Conflict of interest

The author declares that the research was conducted in the absence of any commercial or financial relationships that could be construed as a potential conflict of interest.

## Publisher's note

All claims expressed in this article are solely those of the authors and do not necessarily represent those of their affiliated organizations, or those of the publisher, the editors and the reviewers. Any product that may be evaluated in this article, or claim that may be made by its manufacturer, is not guaranteed or endorsed by the publisher.

## Footnotes

^1^Throughout this article, I use the terms “scarcity” and “abundance” to refer to both (1) a conceptual framework through which policymakers and industry participants make decisions about how to promote software innovation; and (2) descriptors of the actual amount of software innovation taking place. When using these terms to refer to (1), I have attempted to qualify them as “concepts” or “mindsets.
